# Bioactive potential of endophytic fungus *Chaetomium globosum* and GC–MS analysis of its responsible components

**DOI:** 10.1038/s41598-020-75722-1

**Published:** 2020-11-02

**Authors:** Navdeep Kaur, Daljit Singh Arora, Namarta Kalia, Manpreet Kaur

**Affiliations:** 1 Department of Microbiology, Guru Nanak Dev University, Amritsar, Punjab 143005 India; 2 Department of Molecular Biology and Biochemistry, Guru Nanak Dev University, Amritsar, Punjab 143005 India; 3 Department of Human Genetics, Guru Nanak Dev University, Amritsar, 143005 India

**Keywords:** Cancer, Drug discovery, Microbiology, Medical research

## Abstract

The recent exploration of various medicinal plants for bioactive potential has led to the growing interest to explore their endophytes for such bioactive potential which may turn out to be better option than the plants. In the present study, *Chaetomium globosum*, an endophytic fungus isolated from *Moringa oleifera* Lam has been explored for its various biological activities. The chloroformic extract of *C. globosum* showed good antimutagenicity against the reactive carcinogenic mutagen, 2-aminofluorene (2-AF) in Ames test. The antiproliferative activity against various cell lines such as HCT-15, HeLa and U87-MG was found to be dose dependent and the viability reduced to 9.26%, 15.7% and 16.3%, respectively. Further, the chloroformic fungal extract was investigated for free radical scavenging activity using 2, 2-diphenyl-1-picrylhydrazyl (DPPH) and 2,2′-azino-bis(3-ethyl-benzthiazolin-6-sulfonic acid) assay which showed the IC_50_ value of 45.16 µg/ml and 50.55 µg/ml, respectively. The fungal extract also showed good ferric reducing power. Total phenolic and flavonoid content was found to be in linear relationship with the antioxidant potential of the fungal extract. High performance liquid chromatography showed the presence of phenolics which may help to combat the free radicals. The presence of various bioactive compounds was analysed by GC–MS which endorsed *Chaetomium globosum* to be a promising candidate for drug development.

## Introduction

The use of synthetic drugs for various diseases may cause many side effects and sometimes resistance, which justifies the need to find out novel and effective bioactive agents with better mode of action. Many mutagenic and carcinogenic agents are also present in the environment which are responsible for pathogenesis of degenerative diseases^1,2^. These agents trigger the uncontrolled production of reactive oxygen species (ROS) which ultimately damage DNA, proteins etc. This oxidative stress causes major tissue injury in the body which leads to cancer, heart attacks and many other diseases^3^. Therefore, there is a need to explore the untapped resources for the bioactive compounds of pharmaceutical importance.

The traditional medicinal plants are known to produce various compounds responsible for different bioactivities^4,5^. However, the bioactive compounds from the plant associated microbes have not yet been fully explored^6^. Endophytes are microorganisms that colonize the internal tissues of plants without causing any harm to their host and protect them in adverse conditions by producing secondary metabolites. It might be possible that endophytes and their host plant may produce similar bioactive compounds^7^. Therefore, endophytes have been drawing the attention for their metabolites which may turn out to be better than plant metabolites. Endophytic fungi from medicinal plants have thus proven to be a rich source of bioactive metabolites having antimicrobial, antioxidant and anticancer activities^8–11^.Thus, endophytic communities existing in the tissues of living plants are potential resources of novel natural products for exploitation by the pharmaceutical industry.

*Moringa oleifera* commonly known as ‘drumstick tree’ a magic plant, possesses various bioactivities such as antimicrobial, antitumor, antioxidant^12–14^. However, there are less data on the endophytic fungi isolated from this plant. This is apparently the first report on the isolation of *Chaetomium globosum* as endophytic fungus from *Moringa oleifera* where its chloroformic extract containing various secondary metabolites has been studied for antimutagenic, antioxidant and antiproliferative activities. Further, it has been assessed for the presence of various bioactive compounds by GC- MS which might be responsible for such activities.

## Results

### Isolation of fungal endophyte

The molecular identification of endophytic fungal isolate (DSE 72) was done by National Fungal Culture Collection of India (NFCCI), Agharkar Research Institute, Pune, India; it was identified as *Chaetomium globosum.* The sequence obtained was deposited in Genbank under the accession number (MN416318). This fungus has been reported to possess a good antimicrobial activity in our previous study^15^.

### Antimutagenic effect of chloroformic fungal extract

The chloroformic fungal extract showed significant inhibition against the mutagenicity of S9-dependent mutagen, 2-aminofluorene (2-AF). In case of co- incubation, inhibitory effect ranged from 21.9 to 67.1% with IC_50_ 0.79 mg/ml while in case of pre incubation mode, it ranged from 30.4 -70% with IC_50_ 0.35 mg/ml. This demonstrated good inhibitory effect in case of pre incubation as compared to co incubation. The chloroformic extract was found to be neither mutagenic nor toxic to the *Salmonella typhimurium* strain as the number of colonies was comparable to spontaneous mode which did not contain any mutagen. A significant difference (*p* < 0.05) was observed between the various concentrations as revealed by one way ANOVA followed by post hoc Tukey’s t-test (Table 1).

**Table 1:** Antimutagenic effect of chloroformic extract of *C. globosum* on the mutagenicity induced by S9-dependent mutagen (2-AF).

Treatment	Concentration of CFE (mg/ml)	No of colonies Mean ± SE	Percent inhibition (%)
Spontaneous		43 ± 1.52	NA
Positive control (2-AF)		1516.6 ± 13.98	NA
Negative control (without mutagen)	0.05	41.66 ± 2.60	NA
	0.1	31.33 ± 1.45	NA
	0.25	25.66 ± 0.88	NA
	0.5	18.66 ± 0.88	NA
	1	17.66 ± 0.33	NA
Co- incubation	0.05	1193.3 ± 11.0^e^	21.9
	0.1	985.3 ± 3.75^d^	35.7
	0.25	879.3 ± 3.17^c^	42.7
	0.5	691 ± 8.62^b^	55
	1	510.3 ± 3.48^a^	67.1
Pre- incubation	0.05	1067 ± 5.19^e^	30.4
	0.1	880 ± 8.88^d^	42.8
	0.25	765 ± 4.72^c^	50.4
	0.5	600 ± 8.76^b^	61.2
	1	467.3 ± 2.90^a^	70

Values are given as mean ± SE. Different letters (a to e) between the columns are significantly different (Tukey’s test, *p* ≤ 0.05).

### Anti-proliferative assay

**Figure 1:**
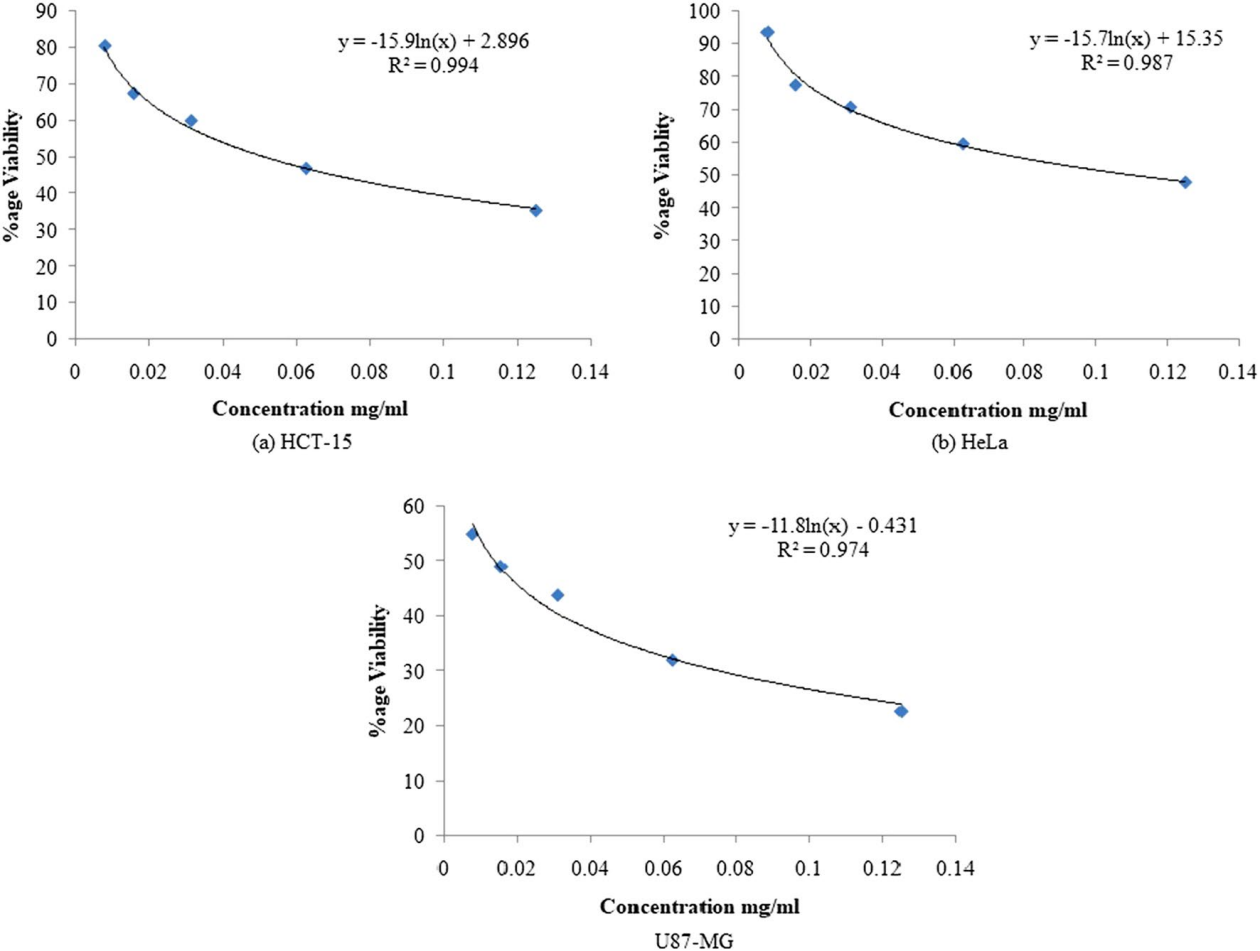
Anti-proliferative activity of chloroformic extract of *Chaetomium globosum* against different cell lines.

The chloroformic fungal extract was assayed for antiproliferative activity against three cancer cell lines; HCT- 15 (colorectal adenocarcinoma), HeLa (cervical cell line), U87-MG (human glioblastoma) by MTT assay. The chloroformic fungal extract showed the dose-dependent anti-proliferative activity against all the tested cancer cell lines. In case of HCT- 15 cell line, the viability reduced to 9.26% at the highest concentration of chloroformic fungal extract i.e. 1 mg/ml and the 50% inhibitory concentration (IC_50_) was found to be 0.051 mg/ml. In case of HeLa cell line, the viability at its highest concentration was found to be 15.7% with IC_50_ value of 0.11 mg/ml and at its lowest concentration the viability was 93.8% whereas in case of U87-MG the viability reduced to 16.3% with IC_50_ value of 0.014 mg/ml. Thus, the chloroformic fungal extract showed potent antiproliferative activity against all the cell lines tested (Fig. 1).

### Antioxidant potential of chloroformic fungal extract

#### DPPH (2, 2-diphenyl-1-picrylhydrazyl) radical scavenging activity

**Figure 2:**
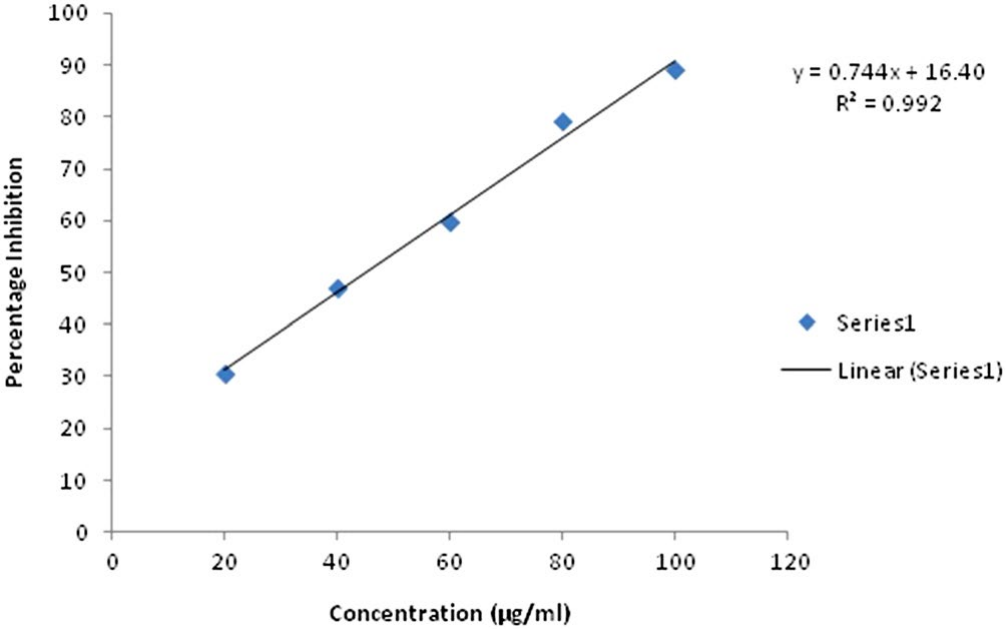
DPPH radical scavenging activity of chloroformic extract of *C. globosum.*

DPPH assay is an excellent method to estimate the potential of antioxidant compounds to scavenge free radicals. When DPPH accepts the hydrogen atom from the antioxidants, discoloration of the molecule takes place. The chloroformic extract tested at the concentration range of 20–100 µg/ml, exhibited 30.59–89% DPPH scavenging activity. The percentage inhibition at the lowest concentration of 20 µg/ml was 30.59%, which significantly increased with the increase in concentration of chloroformic extract. Ascorbic acid as control showed 4.77–75.05% scavenging activity at a concentration range of 2–10 µg/ml. The linear plot obtained at different concentrations of chloroformic fungal extract followed the equation y = 0.744x + 16.40, R^2^ = 0.992 and the 50% inhibitory concentration (IC_50_ value) was found to be 45.16 µg/ml. (Fig. 2).

#### ABTS (2, 2′-azino-bis (3-ethylbenzothiazoline-6-sulphonic acid) radical scavenging activity

**Figure 3:**
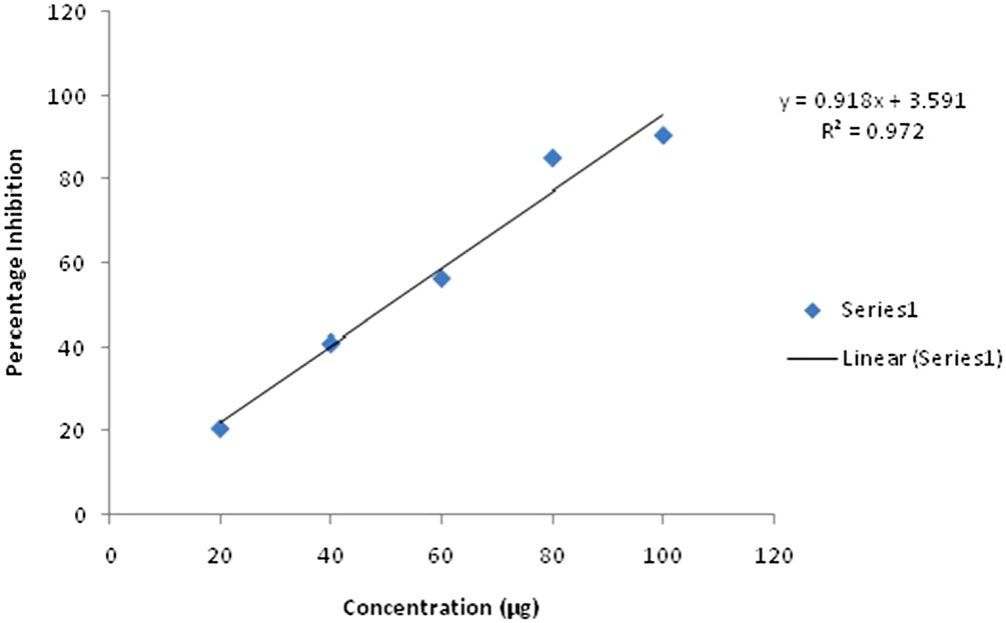
ABTS radical scavenging activity of chloroformic extract of *C. globosum.*

ABTS assay was another method used for estimation of antioxidant potential. This is also a discoloration assay in which antioxidants scavenge the free radicals of ABTS^+^, produced during the reaction of ABTS and potassium persulfate. The chloroformic fungal extract showed significant scavenging activity of 20.6- 90.5% at the concentration range of 20–100 µg/ml. At a low concentration of 20 µg/ml, the percentage inhibition was 20.6 which increased to 41% at a concentration of 40 µg/ml and further increased to 90.5 at its highest concentration of 100 mg/ml. Whereas ascorbic acid showed the inhibition of 31.88- 91.44% at a concentration of 2 to 10 µg. Thus it showed dose dependent scavenging activity and gave the linear equation with IC_50_ value of 50.55 µg/ml (Fig. 3).

#### Ferric reducing antioxidant power (FRAP)

FRAP assay estimates the reducing potential of an antioxidant, reacting with a ferric tripyridyltriazine (Fe3+ -TPTZ) complex and producing a blue coloured ferrous tripyridyltriazine (Fe2+ -TPTZ). The ferric reducing power of the chloroformic fungal extract was calculated from the linear calibration curve of FeSO_4_ and the reducing power was expressed as mM FeSO_4_ equivalents/mg of the fungal extract. The chloroformic extract showed the reducing potential of 86.6 ± 0.032 mM FeSO_4_ equivalents/mg. The results showed the linear relationship with the concentration of the chloroformic extract used.

#### Total phenolic and flavonoids contents

Phenolic compounds are predominantly responsible for the antioxidant potential of microbial extracts. Thus, total phenolic and flavonoid content present in the chloroformic fungal extract is directly proportional to its antioxidant activity. The phenolic content of the chloroformic extract of the fungus was found to be 18.12 ± 0.037 mg GAE/100 mg. The values have been expressed as mg gallic acid equivalents (GAE)/100 mg of chloroformic fungal extract and the calibration curve of gallic acid followed the linear equation of 2.87x + 0.025, R^2^ = 0.99. On the other hand total flavonoid content was 1.862 ± 0.0265 mg quercetin equivalents/100 mg of chloroformic fungal extract. The calibration curve of quercetin followed the linear equation of 21.76x + 0.082, R^2^ = 0.96.

#### Determination of phenolic compounds by ultra high performance liquid chromatography (UHPLC)

The evaluation of various phenolic compounds of the chloroformic extract of *C. globosum*, was carried out by UHPLC. On comparing with standards, phenolic compounds under the curve with different retention times were found to be catechin, chlorogenic acid, umbelliferone, coumaric acid and kaempferol where catechin was in the highest concentration (464.272 mg/l) and coumaric acid showed the least concentration (0.234 mg/l) (Table 2).

**Table 2:** Major phenolic compounds identified in chloroformic extract of *C. globosum.*

Peak	Name	Retention time (in min)	Concentration (in mg/l)
1	Catechin	3.832	464.272
2	Chlorogenic acid	5.058	13.524
3	Caffeic acid	6.815	52.925
4	Umbelliferone	9.293	3.230
5	Coumaric acid	10.204	0.234
6	Kaempferol	17.472	40.250

#### Identification of the non polar bioactive compounds by GC- MS

**Figure 4:**
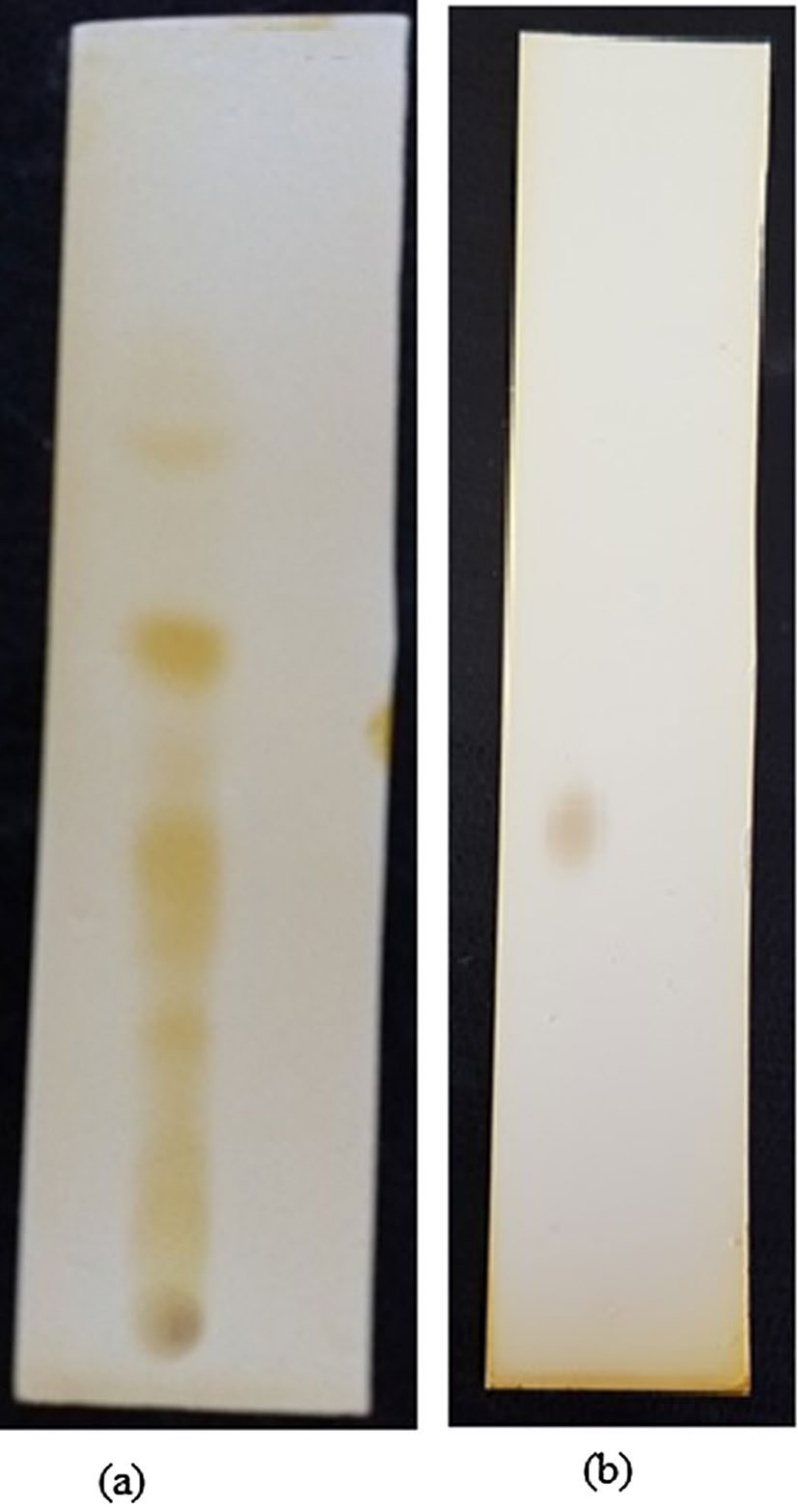
TLC pattern of *C. globosum* (**a**) Crude extract (**b**) Active fraction.

**Figure 5:**
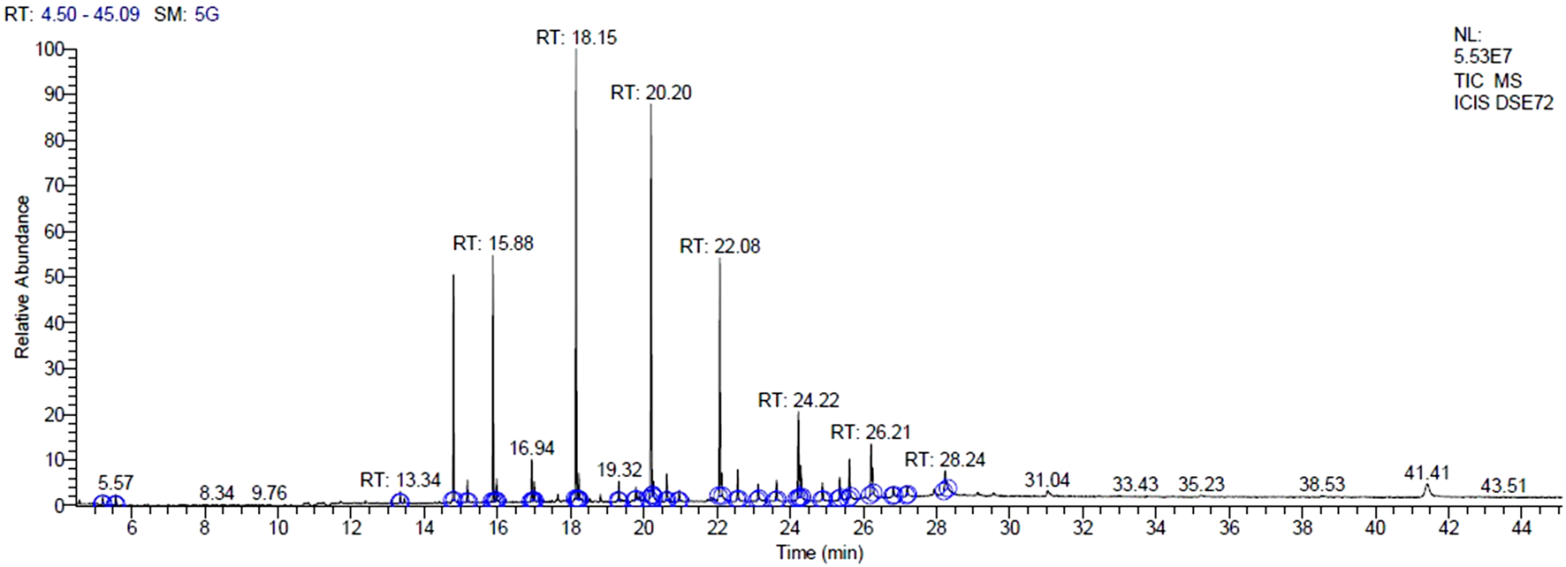
GC–MS chromatogram of compounds from endophytic fungus *C. globosum.*

The fraction obtained from column chromatography showing single band on TLC (Fig. 4) was subjected to HPLC and further analysed for GC–MS which revealed the presence of various compounds with corresponding peaks at different retention times (Fig. 5). Thirty compounds were detected in GC–MS, out of which five were the major compounds namely phenol, 2,4 bis (1,1dimethylethyl); E-14-hexadecenal; 10-heneicosene (c,t); 3- eicosene and 1-heneicosanol (Table 3).

**Table 3:** Bioactive compounds detected in GC–MS analysis of *C. globosum.*

Peak	RT (min)	Compounds	Molecular Formula	Area%
1	14.80	Phenol, 2,4bis(1,1dimethylethyl)	C_14_H_22_O	9.26
2	15.88	E-14-Hexadecenal	C_16_H_30_O	9.95
3	18.15	10-Heneicosene (c,t)	C_21_H_42_	19.29
4	20.20	3- Eicosene	C_21_H_44_O	17.15
5	22.08	1-Heneicosanol	C_21_H_44_O	11.91

## Discussion

A variety of new drugs have been isolated from the natural sources. Now-a-days, endophytes from medicinal plants are gaining a great interest owing to their versatile applications^16^. The endophytic fungi have been recognized as important and novel resources of natural bioactive products with potential pharmaceutical importance. Since the bioactive compound paclitaxel (taxol) was discovered from the endophytic fungus *Taxomyces andreanae* in 1993^17^, many scientists have renewed their interests in studying fungal endophytes as potential producers of novel and biologically active compounds. In the past two decades, many such compounds with different activities have been successfully discovered from endophytic fungi. These bioactive compounds could be classified as alkaloids, terpenoids, steroids, quinones, lignans, phenols, and lactones^7,18^.

In the current study, *Chaetomium globosum* an endophytic fungus isolated from *Moringa oleifera* has been evaluated for antimutagenic activity by Ames test. The chloroformic fungal extract showed a good inhibitory effect on mutagenesis induced by S9-dependent mutagen 2AF (polyaromatic hydrocarbon; PAH) and the inhibitory activity increased with increasing concentrations. The PAH's are promutagens which get activated to their reactive metabolites that covalently bind to DNA. Cytochrome p450, member of CYP1 family is the most active metabolizing enzyme which is involved in the formation of dihydrodiol epoxides, which can subsequently cause mutations in genes^19^. P-450 enzymes metabolize 2-AF into a reactive carcinogenic ester, N-hydroxyl derivative (2-acetylaminofluorene-N-sulfate), which in turn interacts with guanine residues of DNA, resulting in mutagenicity^20,21^. Therefore, the inhibitory potential of the chloroformic fungal extract can be attributed to its ability to scavenge the free radicals produced during the activation of 2AF^22^.

The increasing risk of multi-drug resistance (MDR), the high cost and the adverse side effects of chemotherapy justify the growing need to develop new chemotherapeutic drugs. Several studies have been reported on endophytes to search for novel and effective drugs for cancer therapy^23,24^. In this study, the chloroformic fungal extract showed significant antiproliferative activity against the HeLa, HCT-15 and U87-MG cell lines with IC_50_ values of 110 µg/ml, 51 µg/ml and 14 µg/ml respectively. In a similar study, on the crude extracts of *Acinetobacter guillouiae* an endophyte of *Crinum macowanii* Baker bulbs showed a good bioactive potential against U87MG glioblastoma cell line with IC_50_ value of 6.25 µg/ml^25^. Another endophytic fungus *Penicillium* sp*.* isolated from *Hopea hainanensis* also displayed good cytotoxicity against HepG2 cell line^27^.

Various metabolic activities lead to the generation of reactive oxygen species (ROS) as well as non radical molecules such as O_2_, H_2_O_2_, and OH− . They can play vital roles in the body, such as induction of programmed cell death and tolerance to various environmental stresses. The excessive production of ROS is harmful for the body and known to cause oxidative stress^27^. The most commonly used methods to find out the antioxidant potential of any compound are DPPH and ABTS free radical scavenging activity. The antioxidant compounds help to prevent cancer, aging, and neurodegenerative processes^28^. In this study, the chloroformic fungal extract showed 30.59–89% and 20.6–90.5% scavenging potential for DPPH and ABTS, respectively. This is in consonance with the earlier observations made by Uzma and Chowdappa^8^ on various endophytic fungi. Phenolics and flavonoids act as primary and secondary antioxidants and help in reducing the lipid peroxidation^29^. The chloroformic extract showed a higher phenolic than the flavonoid content, which might be responsible for good scavenging and reducing power of the extract^30^.

The bioactive components present in the chloroformic extract have been analysed using GC–MS which revealed the presence of various compounds that might be responsible for bioactivities. One of these compounds, i.e. phenol, 2,4 bis (1,1dimethylethyl) isolated from *Vibrio alginolyticus* associated with seaweed *Gracilaria gracilis*, has already been reported for its antibiofilm potential^31^ Similarly other such compound i.e. 7-hexadecenal, (Z) has been reported for antiviral activity^32^. Hexadecenal, 10-heneicosene (c,t), 3- eicosene and 1-heneicosanol compounds have been previously reported in literature^33^ but not studied for their antimutagenic, anticancerous and antioxidant activities which adds further credence to the study. Thus, the study concludes with the observation that the endophytic *Chaetomium globosum* possessed a good bioactive potential and can be used for further development of bioactive drug/s for pharmaceutical and medical applications.

## Methods

### Isolation and identification of endophytic fungus

The endophytic fungus was isolated from the freshly collected seed samples of *Moringa oleifera* Lam plant from the botanical garden of Guru Nanak Dev University, Amritsar India. The samples were processed as discussed earlier^15^. Pure culture was maintained at 4 °C in mineral oil. The molecular identification of the isolate (DSE 72) was done by National Fungal Culture Collection of India (NFCCI), Agharkar Research Institute, Pune, India.

### Growth of *Chaetomium globosum* and extract preparation

Fifty ml of yeast peptone dextrose broth (YPD) was inoculated with two discs (8 mm of size) of *Chaetomium globosum* grown on yeast glucose agar (YGA) plates. The flasks were then incubated for 5 days at 25 °C as stationary cultures. The harvested broth was filtered through Whatman filter paper no. 1. The filtrate so obtained was extracted twice with equal volume of chloroform and the solvent layer was evaporated using rotary evaporator. The dried residue obtained was reconstituted in 30% dimethyl sulfoxide (DMSO) and 27.2 mg/ml stock was used for further experimentation^15^.

### Antimutagenic activity of chloroformic fungal extract

The antimutagenic activity of chloroformic fungal extract has been carried out by Ames test using plate incorporation method^34^. *Salmonella typhimurium* strain TA98 was used, provided by the Institute Pasteur, France. From the above mentioned stock of chloroformic extract, different concentrations (0.05-1 mg) were prepared in 30% DMSO. For positive control, 0.1 ml of overnight grown culture and 0.1 ml of 2 AF (2- aminofluorene) mutagen was added to top agar and poured onto minimal agar plates. To determine the toxicity of the fungal extract, 0.1 ml of the overnight grown culture and 0.1 ml of the chloroformic fungal extract were used. Further, to calculate the percentage inhibition of chloroformic fungal extract, the co- incubation and pre-incubation modes were used where 0.1 ml of overnight bacterial culture, 0.5 ml of S9 mix (Sterile distilled water 16.75 ml, 0.2 M Sodium phosphate buffer (pH 7.4), 0.1 M NADP, 1 M G-6-P, MgCl_2_–KCl, Rat liver S9 (phenobarbitone induced), 0.1 ml of 2-AF and 0.1 ml of chloroformic fungal extract were added into top agar and poured onto the plates.

### Co-incubation mode

In co- incubation mode, the bioantimutagenic efficacy was evaluated where bacterial culture, 2-AF, S9 mix and chloroformic fungal extract were added into the top agar and poured onto minimal glucose agar plates.

### Pre-incubation mode

In this mode, the desmutagenic efficacy was evaluated by using pre-incubation mode of treatment, in which S9 mix, 2-AF and chloroformic fungal extract were pre-incubated for 30 min at 37 °C. The mixture was then added into 2 ml top agar containing 0.1 ml of overnight grown bacterial culture and poured onto the plates. The plates were then incubated at 37 °C for 48 h and the revertant colonies were counted. The experiment was done in triplicates.

### Antimutagenic effect

Antimutagenic activity was determined as percent reduction in the number of revertant colonies as below:

$$ \% {\text{ Antimutagenic}}\;{\text{activity}} = {\text{a}} - {\text{b}}/{\text{a}} - {\text{c }} \times { 1}00 $$

where ‘a’ is number of histidine revertant colonies induced by mutagen alone (2-AF); ‘b’ is number of histidine revertant colonies induced by mutagen in the presence of fungal extract; ‘c’ is the number of histidine revertant colonies induced by fungal extract alone.

### Antioxidant potential of chloroformic fungal extract

#### 2,2-diphenyl-1-picrylhydrazyl (DPPH) radical scavenging activity

DPPH radical scavenging activity of the chloroformic fungal extract was worked out according to Joshi et al.^35^ with slight modifications. A stock solution of DPPH (0.1 mM) was prepared in methanol and kept overnight in the dark at room temperature. The working solution of DPPH was prepared by its stock solution diluting with methanol to an absorbance of 0.9 ± 0.05 at 517 nm. The 100 µl of chloroformic extract with different concentration (20– 100 µg/ml) was mixed separately with 1.9 ml of working DPPH solution and kept in dark for 30 min at room temperature. Absorbance was measured at 517 nm.

#### 2,2′-azino-bis (3-ethylbenzothiazoline-6-sulphonic acid (ABTS) radical scavenging activity

ABTS radical scavenging activity of the chloroformic fungal extract was determined according to Joshi et al.^35^. A stock solution of ABTS was prepared using 5 ml of ABTS solution (7 m/mol) mixed with 88 µl of potassium persulphate solution (140 m/mol). The mixture was placed overnight in the dark at room temperature to generate ABTS cation. For the preparation of the working solution of ABTS, its stock solution was diluted with methanol to an absorbance of 0.7 ± 0.05 at 734 nm. Then, 20 µl of the chloroformic fungal extract was added to 980 µl of working solution of ABTS and incubated for 10 min in dark. The absorbance was read at 734 nm spectrophotometrically.

#### Ferric reducing antioxidant activity (FRAP)

The experiment was performed according to Chanda et al.; Arora and Chandra^36,37^. The FRAP reagent was prepared by mixing 300 m/mol acetate buffer (pH 3.6), 10 mM TPTZ solution in methanol and 20 mM ferric chloride solution in the ratio of 10:1:1. To perform the experiment, 2 ml of FRAP reagent was added to 500 μl of the chloroformic fungal extract and 1 ml of distilled water; the reaction mixture was mixed well and incubated for 30 min at room temperature. Absorbance was taken at 593 nm. Ferrous sulphate (FeSO_4_) was taken as a standard to plot the calibration curve. This linear curve is used to calculate the antioxidant potential of the chloroformic extract to reduce the ferric ions. The values were expressed as mM FeSO4 equivalents/100 mg of the chloroformic fungal extract.

#### Determination of total phenolic contents (TPC)

The total phenolic contents were determined colorimetrically using Folin–Ciocalteau (FC) according to Joshi et al.^35^. Test sample (500 µl) was mixed with 100 µl of the 0.5 N FC reagent and after allowing it to stand for 15 min, 2.5 ml of sodium carbonate was added and mixed completely. The reaction mixture was incubated at room temperature for 30 min and its absorbance was measured at 765 nm. Gallic acid was taken as standard and TPC was expressed as mg of gallic acid equivalent (GAE) per 100 mg of the extract.

#### Determination of total flavonoid contents (TFC)

To estimate the total flavonoids, 125 µl of chloroformic fungal extract was mixed with 50 µl of sodium nitrate (5%) and 75 µl aluminium chloride (10%). The reaction mixture was allowed to stand for 6 min to which 250 µl of 1 M sodium hydroxide was added and further diluted with distilled water to make the volume 10 ml and mixed well. Absorbance was recorded at 510 nm against blank. Quercetin was used as a control^35,38^.

#### Determination of phenolic compounds by ultra- high performance liquid chromatography (UHPLC)

The UHPLC analysis was done on Nexera UHPLC (Shimadzu, Japan) system according to Rani et al.^39^. The system was equipped with microsorb-MV 100-5 C-18 column of dimensions 150 × 4.6 × 5 µm particle size, LC- 30 AD quaternary gradient pump and SPD-M20 A diode array detector (DAD). Seventy percent methanol and water was used as mobile phase at a flow rate of 1 ml/min. The column temperature was maintained at 27 °C and the run time of the sample was 26 min. The compounds were identified at 280 nm by comparison of their retention time with available standards.

#### Anti-proliferative activity of chloroformic fungal extract

The cancer cell lines used in the study i.e. HeLa (cervical cell line), HCT-15 (colorectal adenocarcinoma) and U87-MG (human glioblastoma), were procured from National Cell Center, Pune, India. The cell lines were cultured as discussed previously^40^. The anti-proliferative activity of chloroformic fungal extract was determined using 3-(4,5-dimethylthiazol-2-yl)-2,5-diphenyl tetrazolium bromide (MTT) assay^41^. To perform the MTT assay, 100 µl of the cell suspension of different cell lines, having 4 × 10^5^cells/ml was dispensed in each well of the 96-well plate and incubated in CO_2_ incubator at 37 °C. After 24 h, 100 µl of different concentrations of the chloroformic fungal extract were added to the wells and again incubated for 24 h. After incubation the supernatant was discarded and 100 µl of MTT was added to the wells and incubated further for 4 h. The wells were then decanted and 100 µl of dimethyl sulfoxide was added to each well. The absorbance was taken at 570 nm on the micro plate reader. The percentage of proliferation of cells was determined according to the formula given below:

$$ {\text{Percentage}}\;{\text{of}}\;{\text{proliferation}} = \left( {{\text{Absorbance}}\;{\text{of}}\;{\text{treated}}\;{\text{wells}}/\;{\text{Absorbance}}\;{\text{of}}\;{\text{control}}\;{\text{wells}}\; \times \;{1}00} \right) $$

#### Column chromatography for isolation of bioactive compounds

For the extraction of the bioactive compounds, fifty ml of YPDS (yeast extract peptone dextrose starch) broth, taken in 250 ml conical flasks, were inoculated with two discs (8 mm of size) of the fungal culture grown on YGA plates. Incubation was carried out for 5 days as stationary culture. The culture broth obtained from different flasks was pooled and extracted with equal volume of chloroform and then solvent was evaporated using rotary evaporator. Two litres of broth yielded 140 mg of solid residue. Thereafter, it was subjected to column chromatography using silica gel (120–200 mesh size, column 18 mm × 300 mm; Hi-media) packed and pre-equilibrated with chloroform. The column was first eluted with equilibration solvent i.e. chloroform (two bed volumes) followed by linear gradients of chloroform: ethyl acetate (100:0, 90:10, 80:20, 70:30, 60:40, 50:50, 40:60, 30:70, 20:80, 10:90, 0:100) at a flow rate of 1 ml/min). Different fractions, of 25 ml each, were collected and after concentration, were subjected to thin layer chromatography. Chloroform;˗ethyl acetate (3:7) was used as screening system to develop the chromatograms which were observed under UV light (254 and 365 nm) and in iodine chamber. Fractions which showed similar TLC pattern were pooled and concentrated again. Further, the HPLC analysis was carried out.

The most active fraction showing a single band on thin layer chromatography (TLC) was further subjected to high pressure liquid chromatography (HPLC) using (Shimadzu Nexera system, USA) to see the purity of active fraction. Acetonitrile (40%) was used as the mobile phase at a flow rate of 3 ml min^−1^ and injection volume of 300 µl at a temperature of 45 °C. The detections were monitored at 280 nm^42^.

#### GC–MS analysis

The GC–MS analysis of the collected fraction was carried out using Thermo Trace 1300GC coupled with Thermo TSQ 800 Triple Quadrupole MS with column BP 5MS (30 m × 0.25 mm, 0.25 µm). The instrument was set to an initial temperature of 60 °C, and maintained at this temperature for 3 min. Then the oven temperature was raised up to 280 °C, at an increase rate of 15 °C min^-1^ and maintained for 19 min. Injection port temperature was ensured at 260 °C and Helium flow rate as 1 ml min^-1^. The ionization voltage was 70 eV. The samples were injected in split mode as 10:1. Mass spectral scan range was set at 50–650 (m/z)^43^. Using computer searches on a NIST Ver.2.1 MS data library and by comparing the spectrum obtained through GC–MS, compounds present in the extract were identified.

### Informed consent

This article does not contain any studies with human participants performed by any of the authors, so the consent to participate is not applicable.

## References

[1] Andreassi MG, Botto N, Colombo MG, Biagini A, Clerico A (2000). Genetic instability and atherosclerosis: can somatic mutations account for the development of cardiovascular diseases?. Environ. Mol. Mutagen.

[2] Słoczyńska K, Powroźnik B, Pękala E, Waszkielewicz AM (2014). Antimutagenic compounds and their possible mechanisms of action. J. Appl. Genet..

[3] Perron NR, Hodges JN, Jenkins M, Brumaghim JL (2008). Predicting how polyphenol antioxidants prevent DNA damage by binding to iron. Inorg. Chem..

[4] Reyes-López M (2005). The amoebicidal aqueous extract from *Castela texana* possesses antigenotoxic and antimutagenic properties. Toxicol. In Vitro.

[5] Cariño-Cortés R, Hernández-Ceruelos A, Torres-Valencia JM, González-Avila M, Arriaga-Alba M, Madrigal-Bujaidar E (2007). Antimutagenicity of *Stevia pilosa* and *Stevia eupatoria* evaluated with the Ames test. Toxicol. In Vitro.

[6] Gunatilaka AL (2006). Natural products from plant-associated microorganisms: distribution, structural diversity, bioactivity, and implications of their occurrence. J. Nat. Prod..

[7] Zhang HW, Song YC, Tan RX (2006). Biology and chemistry of endophytes. Nat. Prod. Rep..

[8] Uzma F, Chowdappa S (2017). Antimicrobial and antioxidant potential of endophytic fungi isolated from ethnomedicinal plants of Western Ghats Karnataka. J. Pure. Appl. Microbiol..

[9] Tan RX, Zou WX (2001). Endophytes: a rich source of functional metabolites. Nat. Prod. Rep..

[10] Cui JL, Guo TT, Ren ZX, Zhang NS, Wang ML (2015). Diversity and antioxidant activity of culturable endophytic fungi from alpine plants of Rhodiola crenulata, *R. angusta* and *R. sachalinensis*. PloS ONE.

[11] Wu Y, Girmay S, da Silva VM, Perry B, Hu X, Tan GT (2015). The role of endophytic fungi in the anticancer activity of *Morinda citrifolia* Linn. (Noni). Evid. Based Complement. Altern. Med..

[12] Onsare JG, Kaur H, Arora DS (2013). Antimicrobial activity of *Moringa oleifera* from different locations against some human pathogens. J. Med. Plants Res..

[13] Paliwal R, Sharma V, Pracheta SS, Yadav S, Sharma SH (2011). Anti-nephrotoxic effect of administration of *Moringa oleifera* Lam. in amelioration of DMBA-induced renal carcinogenesis in Swiss albino mice. Bio. Med..

[14] Sreelatha S, Padma PR (2009). Antioxidant activity and total phenolic content of *Moringa oleifera* leaves in two stages of maturity. Plant Food Hum. Nutr..

[15] Arora DS, Kaur N (2019). Antimicrobial potential of fungal endophytes from *Moringa oleifera*. Appl. Biochem. Biotech..

[16] Janakiraman N, Johnson M, Sahaya Sathish S (2012). GC-MS analysis of bioactive constituents of *Peristrophe bicalyculata* (Retz.) Nees. (Acanthaceae). Asian Pac. J. Trop. Biomed..

[17] Stierle A, Strobel G, Stierle D (1993). Taxol and taxane production by *Taxomyces andreanae*, an endophytic fungus of Pacific yew. Science.

[18] Xu L, Zhou L, Zhao J, Jiang W (2008). Recent studies on the antimicrobial compounds produced by plant endophytic fungi. Nat. Prod. Res. Dev..

[19] Kondraganti SR, Fernandez-Salguero P, Gonzalez FJ, Ramos KS, Jiang W, Moorthy B (2003). Polycyclic aromatic hydrocarbon inducible DNA adducts: evidence by 32P-postlabeling and use of knockout mice for Ah receptor-independent mechanisms of metabolic activation *in vivo*. Int. J. Cancer.

[20] Miller JA (1970). Carcinogenesis by chemicals: an overview—GHA clowes memorial lecture. Cancer Res..

[21] DeBaun JR, Smith JY, Miller EC, Miller JA (1970). Reactivity *in vivo* of the carcinogen N-hydroxy-2-acetylaminofluorene: increase by sulfate ion. Science.

[22] Phadungkit M, Somdee T, Kangsadalampai K (2012). Phytochemical screening, antioxidant and antimutagenic activities of selected Thai edible plant extracts. J. Med. Plants Res..

[23] Zhan J, Burns AM, Liu MX, Faeth SH, Gunatilaka AA (2007). Search for cell motility and angiogenesis inhibitors with potential anticancer activity: Beauvericin and other constituents of two endophytic strains of *Fusarium oxysporum*. J. Nat. Prod..

[24] Nascimento AMD (2012). Bioactive extracts and chemical constituents of two endophytic strains of *Fusarium oxysporum*. Rev. Bras. Farmacogn..

[25] Sebola TE, Uche-Okereafor NC, Tapfuma KI, Mekuto L, Green E, Mavumengwana V (2019). Evaluating antibacterial and anticancer activity of crude extracts of bacterial endophytes from *Crinum macowanii* Baker bulbs. Microbiol. Open.

[26] Wang FW, Hou ZM, Wang CR, Li P, Shi DH (2008). Bioactive metabolites from *Penicillium* sp., an endophytic fungus residing in Hopea hainanensis. World J. Microb. Biot..

[27] Sharma P, Jha AB, Dubey RS, Pessarakli M (2012). Reactive oxygen species, oxidative damage, and antioxidative defense mechanism in plants under stressful conditions. J. Bot..

[28] Xing R, Yu H, Liu S, Zhang W, Zhang Q, Li Z, Li P (2005). Antioxidant activity of differently regioselective chitosan sulfates *In vitro*. Bioorg. Med. Chem..

[29] Pawle G, Singh SK (2014). Antioxidant potential of endophytic fungus *Colletotrichum* species isolated from *Polygala elongate*. Int. J. Pharma Bio. Sci..

[30] Govindappa M, Channabasava R, Sunil Kumar KR, Pushpalatha KC (2013). Antioxidant activity and phytochemical screening of crude endophytes extracts of *Tabebuia argentea* Bur. & K. Sch. Am. J. Plant Sci..

[31] Padmavathi AR, Abinaya B, Pandian SK (2014). Phenol, 2, 4-bis (1, 1-dimethylethyl) of marine bacterial origin inhibits quorum sensing mediated biofilm formation in the uropathogen *Serratia marcescens*. Biofouling.

[32] Devakumar J, Keerthana VS, Sudha SS (2017). Identification of bioactive compounds by gas chromatography-mass spectrometry analysis of *Syzygium jambos* (L.) collected from Western Ghats region Coimbatore, Tamil Nadu. Asian J. Pharm. Clin. Res..

[33] Dong-Mei W, Lin-Fang H (2012). Composition of volatile oil from the leaves of *Uncaria sessilifructus Roxb*. J. Appl. Pharm. Sci..

[34] Maron DM, Ames BN (1983). Revised methods for the *Salmonella* mutagenicity test. Mut. Res..

[35] Joshi R, Rana A, Gulati A (2015). Studies on quality of orthodox teas made from anthocyanin-rich tea clones growing in Kangra valley India. Food Chem..

[36] Chanda S, Dave R (2009). *In vitro* models for antioxidant activity evaluation and some medicinal plants possessing antioxidant properties: An overview. Afr. J. Microbiol. Res..

[37] Arora DS, Chandra P (2010). Assay of antioxidant potential of two *Aspergillus* isolates by different methods under various physio-chemical conditions. Braz. J. Microbiol..

[38] Sharma A, Mahajan H, Dwivedi JP, Gupta M (2015). Optimization of nutritionally enriched mango bar using response surface methodology. J. Food. Meas. Charact..

[39] Rani R, Arora S, Kaur J, Manhas RK (2018). Phenolic compounds as antioxidants and chemopreventive drugs from *Streptomyces cellulosae* strain TES17 isolated from rhizosphere of *Camellia sinensis*. BMC Complement Altern. Med..

[40] Kaur N, Arora DS, Kalia N, Kaur M (2020). Antibiofilm, antiproliferative, antioxidant and antimutagenic activities of an endophytic fungus *Aspergillus fumigatus* from *Moringa oleifera*. Mol. Biol. Rep..

[41] Mosmann T (1983). Rapid colorimetric assay for cellular growth and survival: application to proliferation and cytotoxicity assays. J. Immun. Methods.

[42] Mao Z, Luo R, Luo H, Tian J, Liu H, Yue Y, Wang M, Peng Y, Zhou L (2014). Separation and purification of bioactive botrallin and TMC-264 by a combination of HSCCC and semi-preparative HPLC from endophytic fungus *Hyalodendriella* sp. Ponipodef 12. World J. Microb. Biot..

[43] Senthilkumar N, Murugesan S, Babu DS, Rajeshkannan C (2014). GC-MS analysis of the extract of endophytic fungus, *Phomopsis* sp. isolated from tropical tree species of India, *Tectona grandis* L.. Int. J. Innov. Res. Sci. Eng. Tech..

